# Molecular characterization of a novel botourmiavirus infecting *Leptosphaeria biglobosa*

**DOI:** 10.1007/s00705-026-06757-4

**Published:** 2026-10-07

**Authors:** Nesma M. Samir, Syun-ichi Urayama, Jacob Locke-Gotel, Yong-Ju Huang, Bruce D. L. Fitt, Robert H. A. Coutts, Hiromitsu Moriyama, Ioly Kotta-Loizou

**Affiliations:** 1 Laboratory of Molecular and Cellular Biology, Department of Applied Biological Sciences, Tokyo University of Agriculture & Technology, 3-5-8, Saiwaicho, Tokyo, 183-8509 Fuchu Japan; 2 Department of Botany, Faculty of Science, Mansoura University, Mansoura, 35516 Egypt; 3 Laboratory of Biology for Extreme Molecule, Department of Life and Environmental Sciences, University of Tsukuba, 1-1-1 Tennodai, Tsukuba, 305-8577 Ibaraki Japan; 4 School of Health, Medicine and Life Sciences, University of Hertfordshire, Hatfield, AL10 9AB UK; 5 Department of Life Sciences, Faculty of Natural Sciences, Imperial College London, London, SW7 2AZ UK; 6 Division of Evolution, Infection and Genomics, School of Biological Sciences, University of Manchester, Manchester, M13 9PL UK

## Abstract

The phytopathogenic fungus *Leptosphaeria biglobosa* is the host of diverse mycoviruses. Comprehensive analysis of next-generation sequencing (NGS) data obtained from purified double-stranded RNA (dsRNA) revealed the presence of three single-stranded RNA (ssRNA) molecules. These RNAs, designated ssRNA1, ssRNA2, and ssRNA3, are respectively 2,646, 683, and 575 bp in length. ssRNA1 contains a single open reading frame (ORF) encoding a putative RNA-dependent RNA polymerase (RdRP) and represents the genome of a novel member of the family *Botourmiaviridae*, designated as “Leptosphaeria biglobosa botourmiavirus 3” (LbBoV-3). Conversely, ssRNA2 and ssRNA3 share identical 3′-termini but no other sequence similarity with ssRNA1, lack substantial ORFs, do not possess poly(A) tails, and are proposed to represent satellite-like RNAs associated with LbBoV-3. To our knowledge, LbBoV-3 is the second member of the genus *Magoulivirus* associated with two satellite RNAs, expanding the current understanding of mycoviral diversity associated with this economically important phytopathogenic fungus.

**Supplementary Information:**

The online version contains supplementary material available at https://doi.org/10.1007/s00705-026-06757-4.

Mycoviruses infect a broad range of fungi, including many phytopathogens [1]. Although most known mycoviruses harbor double-stranded RNA (dsRNA) genomes, recent studies have revealed a rapidly increasing diversity of positive-sense single-stranded RNA (+ ssRNA) mycoviruses. Among these, the family *Botourmiaviridae* was established by the International Committee on Taxonomy of Viruses (ICTV) in 2018 to accommodate a distinct group of linear +ssRNA viruses [2]. Currently, mycoviruses within the family *Botourmiaviridae* are assigned to 9 genera, possess mono- or multisegmented +ssRNA genomes and predominantly infect filamentous fungi. Phylogenetically, members of this family are related to viruses belonging to the families *Ourmiaviridae* and *Rhizouliviridae* [3].

In addition to expanding the diversity of botourmiaviruses, recent studies have also identified associated satellite-like RNAs adding another level of complexity. Satellite-like RNAs rely on a replication-competent helper virus for their maintenance, are commonly associated with fungal or plant viruses, and may or may not influence host symptoms [4, 5]. Unlike defective RNAs, which arise from helper virus genomes through deletion and/or rearrangement events, satellite-like RNAs are genetically distinct and do not retain substantial sequence similarity with their helper viruses [6]. Reports of satellite-like RNAs associated with mycoviruses remain scarce, while their evolutionary origin, biological significance, and interactions with helper viruses are still poorly understood.

*Brassica napus* (oilseed rape/canola) represents the most economically valuable species within the genus *Brassica* and is among the most important oilseed crops worldwide, ranking third after palm oil and soybean. *B. napus* has been cultivated in Europe since the early medieval period and its global production has continued to expand, with China, Canada and Europe constituting the principal production regions, resulting in a consistent increase in yield over recent decades [7]. Although of high agronomic importance, *B. napus* is susceptible to several major diseases of global significance, including *Sclerotinia* stem rot, clubroot, and phoma stem canker or blackleg [8]. The latter, caused by the fungal species complex *Leptosphaeria maculans* and *Leptosphaeria biglobosa*, is considered one of the most damaging diseases of rapeseed, leading to average yield losses of 10–15% and, under severe epidemic conditions, losses reaching up to 90% [9, 10].

To date, numerous (+)ssRNA, dsRNA, and (−)ssRNA mycoviruses have been detected in *L. biglobosa*. The (+)ssRNA viruses include members of the families *Mitoviridae*, *Botourmiaviridae*, *Hypoviridae*, and *Flaviviridae*, as well as viruses belonging to the order *Tymovirales* and narna-like viruses within the phylum *Lenarviricota*. In addition, dsRNA viruses belonging to the families *Partitiviridae*, *Quadriviridae*, *Botybirnaviridae*, and *Polymycoviridae*, together with (−)ssRNA viruses assigned to the families *Mymonaviridae*, *Aspiviridae*, and *Discoviridae*, have also been identified [11].

In the present study, we identified an additional (+)ssRNA mycovirus infecting *L. biglobosa* isolate W32, belonging to the genus *Magoulivirus* in the family *Botourmiaviridae* and therefore designated as “Leptosphaeria biglobosa botourmiavirus 3” (LbBoV-3), together with two associated RNAs. We characterized their genome organization, conserved sequence features, and phylogenetic relationships, evaluating the characteristics that support the classification of the two associated RNAs as satellite-like RNAs.

*L. biglobosa* isolate W32 originated from an oilseed rape field in Canada and was obtained from the OREGIN collection, United Kingdom (oregin.org.uk). It was initially cultured on yeast extract–glucose agar (YGA; 0.5% yeast extract, 2% glucose, and 1.5% agar) plates at 25 °C and subsequently sub-cultured in YG broth at 25 °C for 10 days under shaking, as previously described [12]. Total RNA was extracted from 100 mg of fungal mycelia using the RNeasy Plant Mini Kit (QIAGEN, Hilden, Germany) according to the manufacturer’s instructions. Subsequently, dsRNA including genomes and replicative forms of RNA viruses was enriched from the extracted total RNA by cellulose D chromatography (Advantec, Tokyo, Japan) as previously described [13, 14]. Contaminating DNA and ssRNA were removed by treatment with DNase I and S1 nuclease, respectively [14].

Fragmented and primer-ligated dsRNA sequencing (FLDS) libraries were sequenced on an Illumina MiSeq platform using 2 × 300-bp paired-end reads [15]. Raw sequencing reads were processed following the FLDS pipeline [15], including quality filtering, adapter trimming, removal of PhiX, low-complexity, and rRNA-derived reads, followed by *de novo* assembly using CLC Genomics Workbench version 11.0 (Qiagen) with a k-mer size of 39 bp, a bubble size of 500 bp, and read mapping parameters of 0.9 for both length and similarity fractions. The resulting contigs were manually inspected using CLC Genomics Workbench, Genetyx version 14, and Tablet version 1.19.09.03 to verify genome completeness. Selected regions of the viral assemblies were confirmed by Sanger sequencing following cDNA synthesis with random primers and polymerase chain reaction (PCR) amplification using virus-specific primers (Table S1). The terminal sequences were determined by RNA ligase-mediated rapid amplification of cDNA ends (RLM-RACE) [16].

The assembled contigs were searched against the National Centre for Biotechnology Information (NCBI) non-redundant (nr) protein database using the Basic Local Alignment Search Tool (BLAST)x with an E-value threshold of ≤ 1 × 10⁻⁵ to identify virus-related sequences. The complete genome sequence was analyzed using the NCBI Open Reading Frame (ORF) Finder to predict putative ORFs (https://www.ncbi.nlm.nih.gov/orffinder/). Similarity searches of predicted amino acid (aa) sequences were performed using BLASTp, whereas pairwise nucleotide (nt) sequence comparisons were performed using BLASTn [17]. Conserved protein domains were identified using NCBI Conserved Domain Database (CDD) [18]. Potential secondary structures (stem–loop structures) within the 5′- and 3′-untranslated regions (UTRs) were predicted using the MFOLD web server [19]. RNA sequences were folded as linear molecules at the server’s fixed temperature of 37 °C under ionic conditions of 1 M NaCl without divalent ions. The percent suboptimality was set to 5%, with a maximum of 50 predicted foldings. The maximum interior/bulge loop size and maximum asymmetry were each set to 30, whereas all other parameters were left at their default settings.

Multiple sequence alignments for conserved motif analysis were generated using the Multiple Alignment using Fast Fourier Transform (MAFFT) implemented in JalView version 2.11.5.2 [20, 21]. For phylogenetic analysis, aa sequences were aligned using the MUSCLE algorithm implemented in Molecular Evolutionary Genetics Analysis (MEGA) version 12 [22]. Phylogenetic trees were constructed using the neighbor-joining (NJ) method with 1,000 bootstrap replicates. Three members of the family *Ourmiaviridae* were used as outgroups.

Using total RNA sequencing, three distinct ssRNA molecules were identified, one of which represents the LbBoV-3 genome, while the other two are proposed to be satellite-like RNAs. Out of a total of 2,281,346 reads were generated, 11,655 mapped to the LbBoV-3 genome and the two satellite-like RNAs. The average sequencing coverage was 207.6× for LbBoV-3, 744.8× for satellite-like RNA 1, and 599.7× for satellite-like RNA 2. The presence of LbBoV-3 and the two satellite-like RNAs in the original fungal isolate was confirmed by RT-PCR using virus-specific primers (Fig. S1). The complete nucleotide sequences of LbBoV-3 genomic RNA, satellite-like RNA 1, and satellite-like RNA 2 have been deposited in the DNA Data Bank of Japan (DDBJ) under accession numbers LC932353, LC932354, and LC932355, respectively.

LbBoV-3 ssRNA1 is 2,646 nt in length with a GC content of 50.45% (1,335/2,646) and contains a single ORF1 of 1,944 nt spanning positions 290 to 2,233, encoding a putative protein of 647 aa with a predicted *Mr* of 74.6 kDa (Fig. 1A). The genome also includes a 5′ UTR of 289 nt and a 3′ UTR of 413 nt (Fig. 1A). Both the 5′ termini (nt positions 1–49) and the 3′ termini (nt positions 2,606–2,646) were predicted by MFOLD to form three stem-loop structures, with minimum free energy (ΔG) values of − 26.00 and − 9.10 kcal/mol, respectively (Fig. S2). The ability of non-coding regions at both ends of the genome to form one or more stem-loop structures is a common and conserved feature among members of the families *Botourmiaviridae*, *Narnaviridae*, and *Mitoviridae* [23, 24].

**Fig. 1:**
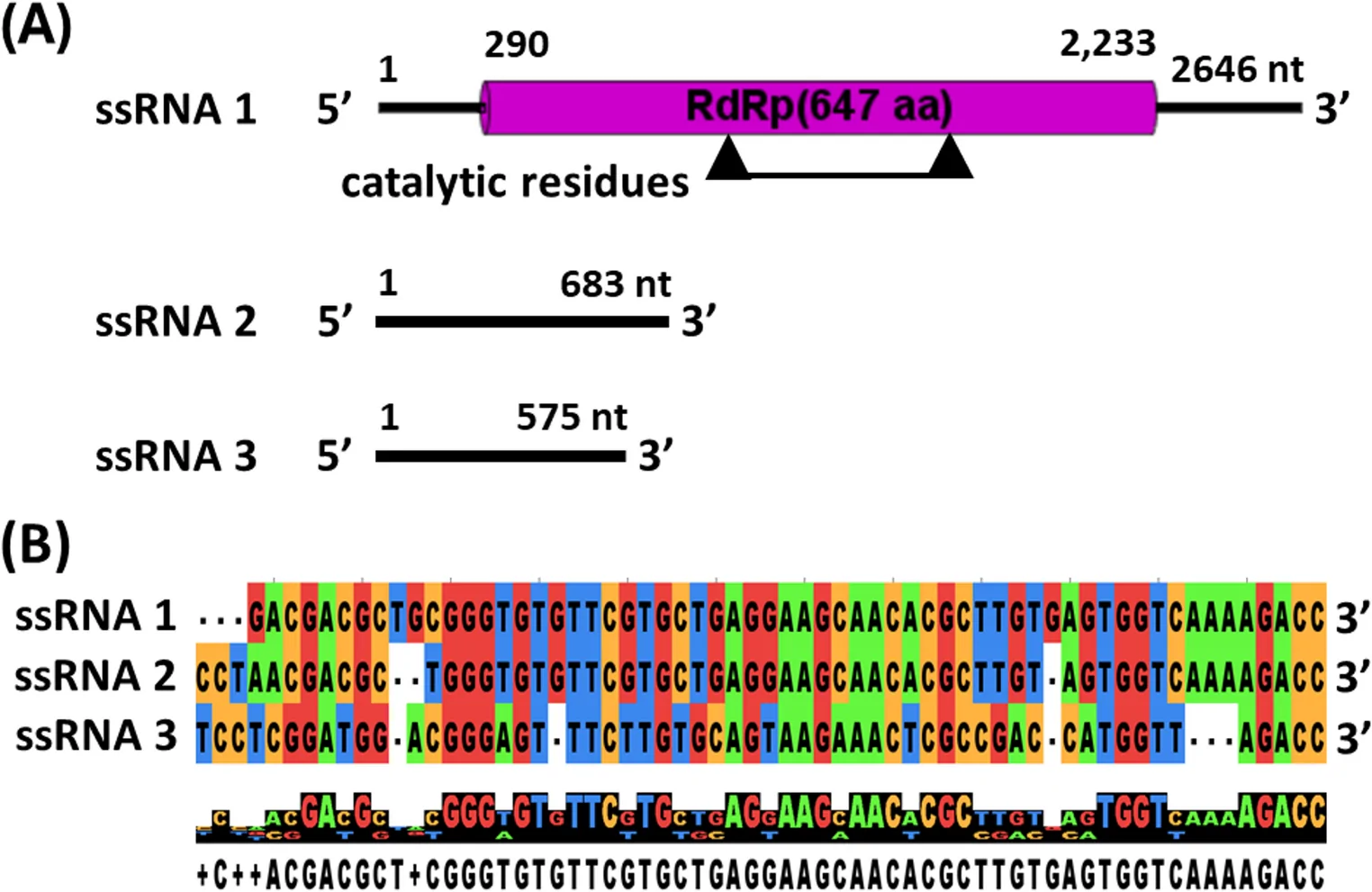
Genomic organisation. (**A**) Schematic diagram of the genome structure of LbBoV-3. The single ORF encoding the putative RdRP (647 aa) is represented by a purple box, while the 5′ and 3′ UTRs are shown as black lines. Conserved catalytic residues within the RdRP are indicated by black triangles. ssRNA2 and ssRNA3 represent satellite-like RNA elements associated with LbBoV-3. (**B**) Comparison of the 3′-terminal sequences of the three ssRNAs. Identical nucleotide regions are conserved at the 3′ ends of all segments

LbBoV-3 RNA-dependent RNA polymerase (RdRP) was most closely related to the RdRPs of some members of the genus *Magoulivirus* in the family *Botourmiaviridae*, as shown by BLASTp (Table S2). Furthermore, a CDD search and a multiple aa sequence alignment confirmed that the protein encoded by LbBoV-3 contains eight typical conserved motifs that are characteristic of the RdRPs of +ssRNA viruses (Fig. S3) [25]. Phylogenetic analysis was performed using the RdRP aa sequence of LbBoV-3 together with 67 representative members of the family *Botourmiaviridae*, encompassing all currently recognized genera. Representative taxa were selected from genera containing numerous members to ensure broad taxonomic coverage, whereas the closest homologs identified by BLASTp were additionally included to improve phylogenetic resolution within the genus *Magoulivirus* (Table S3). As shown in Fig. 2, LbBoV-3 clustered within the *Magoulivirus* clade and formed a strongly supported clade (bootstrap value = 100%) with the unclassified magouliviruses WAK77897.1, WAK77907.1, and WAK77873.1. This phylogenetic placement was consistent with the BLASTp results and with previously reported phylogenetic analyses of members of the family *Botourmiaviridae* [26–28]. Together, these findings support the classification of LbBoV-3 as a novel member of the genus *Magoulivirus*.

**Fig. 2:**
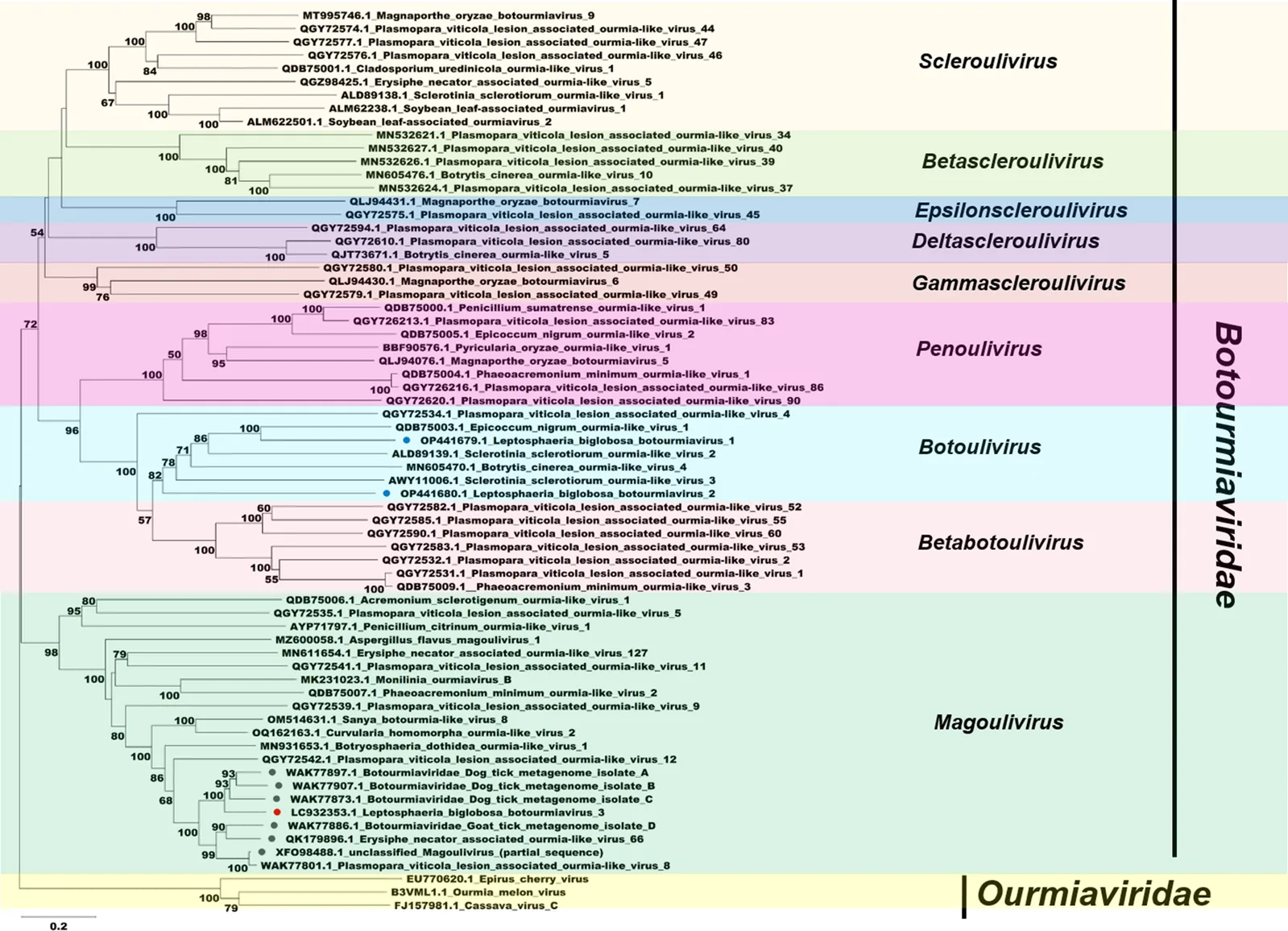
Phylogenetic analysis. Phylogenetic tree constructed using the NJ method based on RdRP aa sequences of LbBoV-3 and representative members of different genera within the family *Botourmiaviridae*. LbBoV-3 is indicated by a red dot and clusters with unclassified members of the genus *Magoulivirus* indicated by grey dots also blue dots indicate previously identified *botourmiaviruses* infecting *L. biglobosa*. Bootstrap analysis was performed with 1,000 replicates, and bootstrap values greater than 50% are shown at the nodes. The scale bar represents 0.20 aa substitutions per site

The full-length cDNAs of ssRNA2 and ssRNA3 are 683 and 575 nt in length, with GC contents of 50.66% and 54.43%, respectively. A single small ORF was predicted in ssRNA2, encoding a 34 aa peptide with an estimated *Mr* of 3.9 kDa, whereas ssRNA3 was predicted to encode a 57 aa peptide with an estimated *Mr* of 6.45 kDa (data not shown). However, sequence analysis revealed that neither predicted peptide showed significant similarity to any known protein. No significant internal nucleotide sequence similarity was noted between either ssRNA2 or ssRNA3 and the LbBoV-3 genome following pairwise BLASTn analysis. The only significant BLASTn hit was restricted to a short region at the 3′ terminus shared between ssRNA2 and the LbBoV-3 genomic RNA (Fig. S4). Furthermore, ssRNA2 and ssRNA3 appear to be dispensable and are proposed to represent satellite-like RNAs associated with LbBoV-3. Nevertheless, their dependence on LbBoV-3 as a helper virus remains to be experimentally verified.

Although two additional botourmiaviruses, Leptosphaeria biglobosa botoulivirus 1 (LbBV1) and Leptosphaeria biglobosa botoulivirus 2 (LbBV2), both belonging to the genus *Botoulivirus*, have recently been identified in *L. biglobosa* [11], LbBoV-3 represents the first member of the genus *Magoulivirus* identified in *L. biglobosa*. Members of this genus have also been identified in several phytopathogenic fungi, including *Botryosphaeria dothidea*, *Magnaporthe oryzae*, *Botrytis cinerea*, and *Phomopsis asparagi* [27, 29–31]. However, most currently recognized members have been characterized only at the genome sequence level, with limited information regarding their biological properties. Among the few biologically characterized members of the genus *Magoulivirus*, Fusarium oxysporum ourmia-like virus 1 (FoOuLV1), infecting *Fusarium oxysporum*, the causal agent of Fusarium wilt, was shown to reduce mycelial growth and virulence, suggesting its potential as a biological control agent [32]. Similarly, Fusarium solani ourmia-like virus 1 (FsoOLV1), another member of the genus *Magoulivirus* infecting *Fusarium solani*, was recently reported to confer hypovirulence and horizontal transmissibility between fungal strains [33]. These findings suggest that at least some *Magoulivirus* members can modulate fungal pathogenicity. Whether LbBoV-3 exerts similar effects on the growth, phenotype, or virulence of *L. biglobosa* remains unknown and warrants further investigation.

One of the most notable findings of the present study is the identification of two RNAs associated with LbBoV-3 that exhibit several genomic characteristics consistent with satellite-like RNAs. Both RNAs are considerably smaller than the genomic RNA of LbBoV-3, contain only small putative ORFs with no detectable similarity to known proteins, lack an RdRP-encoding ORF and poly(A) tails, and possess conserved terminal sequence motifs at both the 5′ and 3′ termini. Furthermore, pairwise BLASTn analysis revealed no significant internal nt sequence similarity between either RNA and the LbBoV-3 genome, with the only significant match restricted to a short region at the 3′ terminus of ssRNA2. These observations distinguish the two RNAs from typical defective RNAs, which generally arise through deletion and/or rearrangement of the helper virus genome while retaining substantial sequence similarity with the parental viral genome [6, 34]. Collectively, these genomic characteristics support the interpretation that ssRNA2 and ssRNA3 are more consistent with satellite-like RNAs than with defective RNAs. Nevertheless, because experimental evidence demonstrating their dependence on LbBoV-3 as a helper virus is currently lacking, they are provisionally designated as satellite-like RNAs.

Recently, Kondo et al. [35]. described two satellite-like RNAs associated with a *Magoulivirus* infecting *Aspergillus luchuensis.* Similar to the present study, these RNAs were identified primarily on the basis of their genomic organization and co-occurrence with the putative helper virus, whereas direct experimental evidence demonstrating helper virus dependence was not available. Although both studies relied primarily on genomic evidence, the occurrence of satellite-like RNAs in two independent *Magoulivirus* systems suggests that these subviral elements may also occur in additional members of this genus. However, further studies involving a broader range of magouliviruses will be required to determine their prevalence, evolutionary origin, and biological significance.

Although the biological significance of fungal satellite-like RNAs remains poorly understood, satellite or satellite-like RNAs have been identified in association with several mycovirus systems infecting filamentous fungi and yeast [36–39]. Available evidence indicates that these subviral elements can influence helper virus biology and, in some virus–host systems, alter fungal phenotypes. For example, a satellite-like RNA associated with Pestalotiopsis fici hypovirus 1 (family *Hypoviridae*) was shown to negatively regulate helper virus accumulation without causing detectable changes in fungal growth or virulence [39]. In another well-characterized fungal virus system, satellite M dsRNAs associated with the Saccharomyces cerevisiae L-A helper virus (family *Totiviridae*) encode killer toxins that confer a competitive ecological advantage to infected yeast cells [37]. Whether the two satellite-like RNAs identified in association with LbBoV-3 (family *Botourmiaviridae*) similarly influence helper virus replication, fungal pathogenicity, or other aspects of the interaction between LbBoV-3 and *L. biglobosa* remains unknown. Future studies, including virus-curing, transfection, and infectivity assays, will be required to determine their biological functions and to experimentally verify their dependence on LbBoV-3 as a helper virus.

Overall, the identification of LbBoV-3 together with two associated satellite-like RNAs expands the currently recognized diversity of members of the genus *Magoulivirus* and provides additional evidence that satellite-like RNAs may constitute a previously underappreciated component of the genomic architecture of botourmiaviruses. Continued characterization of these subviral elements from diverse fungal hosts will improve our understanding of their evolutionary origins, biological functions, and potential roles in shaping virus–fungus interactions.

## Supplementary Information

Below is the link to the electronic supplementary material.

Supplementary Material 1

## Data Availability

The complete nucleotide sequences of LbBoV-3 genomic RNA, satellite-like RNA 1, and satellite-like RNA 2 have been deposited in the DNA Data Bank of Japan (DDBJ) under accession numbers LC932353, LC932354, and LC932355, respectively.
