# Supplementary material for: Molecular characterization of a novel botourmiavirus infecting Leptosphaeria biglobosa
Source: Arch Virol. 2026 Oct 7;171(11):291. doi: 10.1007/s00705-026-06757-4 (PMC13646007; doi:10.1007/s00705-026-06757-4)
Supplement: Supplementary file 1 [file 705_2026_6757_MOESM1_ESM.pdf]

Table S1. Primers used in this study.

Primer name	Primer sequence (5' to 3')	Position (nt)	Product (bp)
**LbBoV-3 RdRP F1**	ATCCGAAGATTCTAGACAAGCCC	11-33	RNA1 (673 bp)
**LbBoV-3 RdRP R1**	CGGAAAGTGATACCTCAAGAGCT	662-684	
**LbBoV-3 RdRP F2**	GGTATTCACTCTACCCACATCGG	841-863	RNA1 (526 bp)
**LbBoV-3 RdRP R2**	GCTCTCTTTTAAGTTTCCCGCTG	1344-1366	
**LbBoV-3 RdRP F3**	CTCAGATCCACTTGCCTTTTTGG	1604-1626	RNA1 (682 bp)
**LbBoV-3 RdRP R3**	GGGACGTTGAAAGTAGGAAGACT	2263-2285	
**LbBoV-3 satRNA1 F**	GGGTAAAGACTCGGCAAAACC	42-62	RNA2 (564 bp)
**LbBoV-3 satRNA1 R**	GGAAAGGTTGTGGGTTCGAATC	584-605	
**LbBoV-3 satRNA2 F**	GGTAACAAGTGAATATGCCAGCG	85-107	RNA3 (486 bp)
**LbBoV-3 satRNA2 R**	CGGGAGTTTCTTGTGCAGTAAGA	530-552	

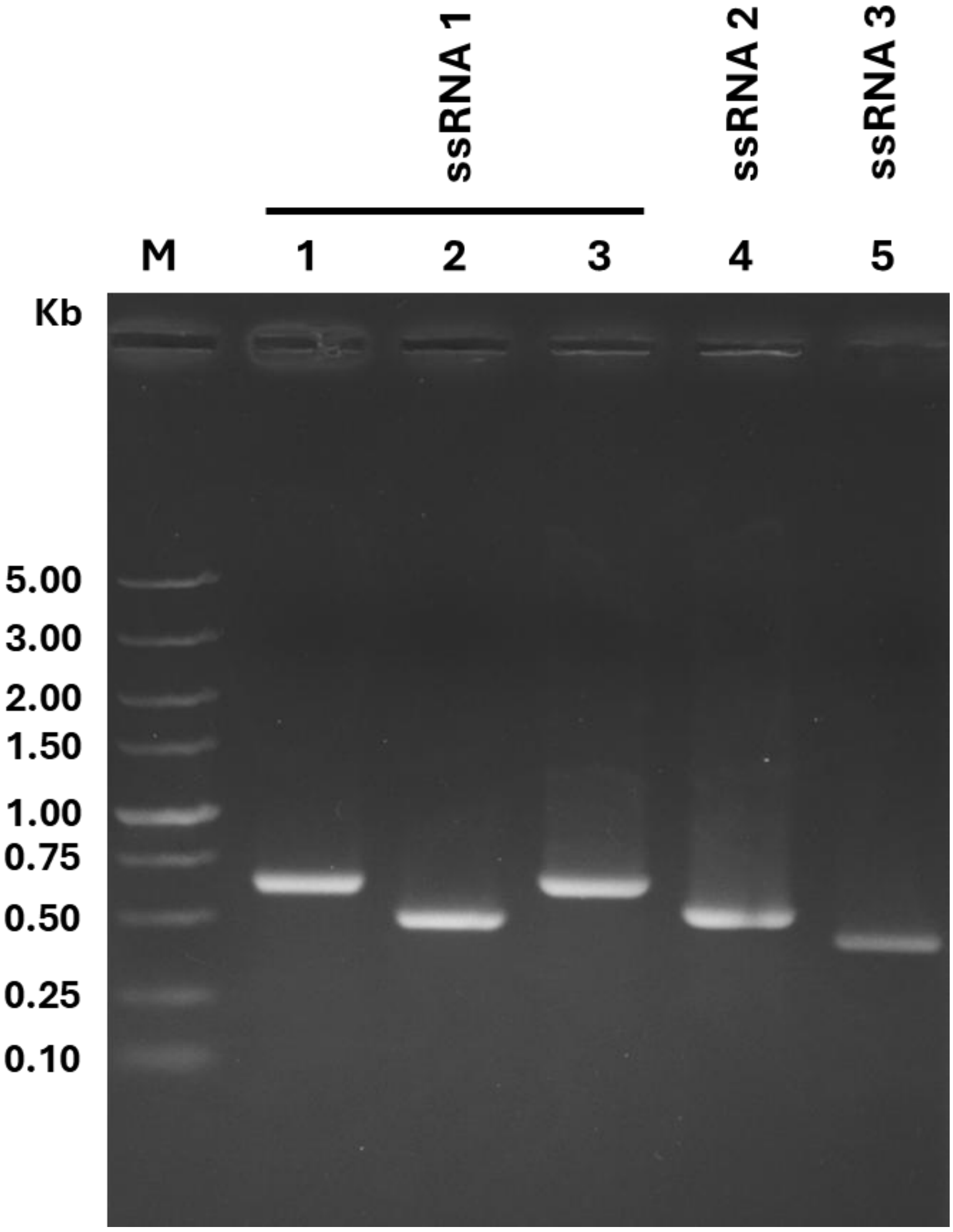

Figure S1. RT-PCR detection of LbBoV-3 and associated satellite-like RNAs. RT-PCR analysis shows the presence of LbBoV-3 in lanes 1–3 using the three primer pairs listed in Table S1, whereas satellite-like RNAs are detected in lanes 4 and 5. Lane M indicates the DNA size marker.

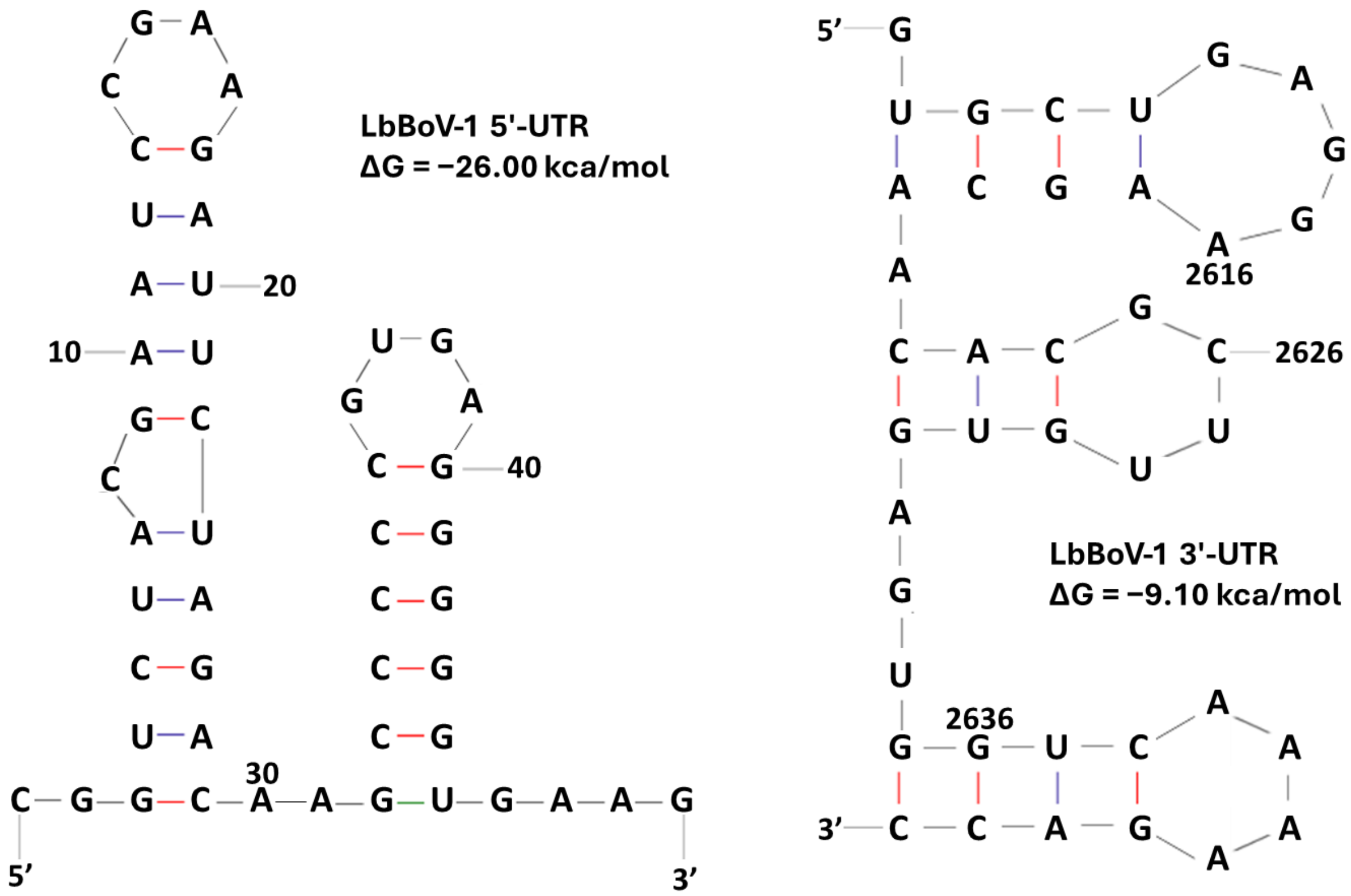

Figure S2. Predicted RNA secondary structures of the LbBoV-3 5' and 3' UTRs. Stem–loop structures were identified at the 5' and 3' termini of the LbBoV-3 genome, with predicted ΔG values of −26.00 and −9.10 kcal/mol, respectively. Short colored lines indicate hydrogen bonds between base pairs (red, G–C pairs; purple, A–U pairs; green, G–U pairs).

Table S2. Established members of the genus *Magulivirus*, family *Botourmiaviridae*, with significant similarity to LbBoV-3.

Accession number	Virus name	Identity	Coverage	E-value
**WAK77897.1**	Dog tick metagenome isolate A	81.63%	98%	0.0
**WAK77907.1**	Dog tick metagenome isolate B	80.72%	98%	0.0
**WAK77873.1**	Dog tick metagenome isolate C	80.09%	99%	0.0
**QK179896.1**	Erysiphe necator associated ourmia-like virus 66 (EnAOLV-66)	73.46%	98%	0.0
**WAK77801.1**	Plasmopara viticola lesion associated ourmia-like virus 8 (PavOLV8)	72.86%	97%	0.0
**WAK77886.1**	*Botourmiaviridae* goat tick metagenome isolate D	72.46%	96%	0.0
**QGY72542.1**	Plasmopara viticola lesion associated ourmia-like virus 12 (PavOLV12)	62.76%	94%	0.0
**MN931653.1**	Botryosphaeria dothidea ourmia-like virus 1 (BdOLV1)	59.05%	93%	0.0

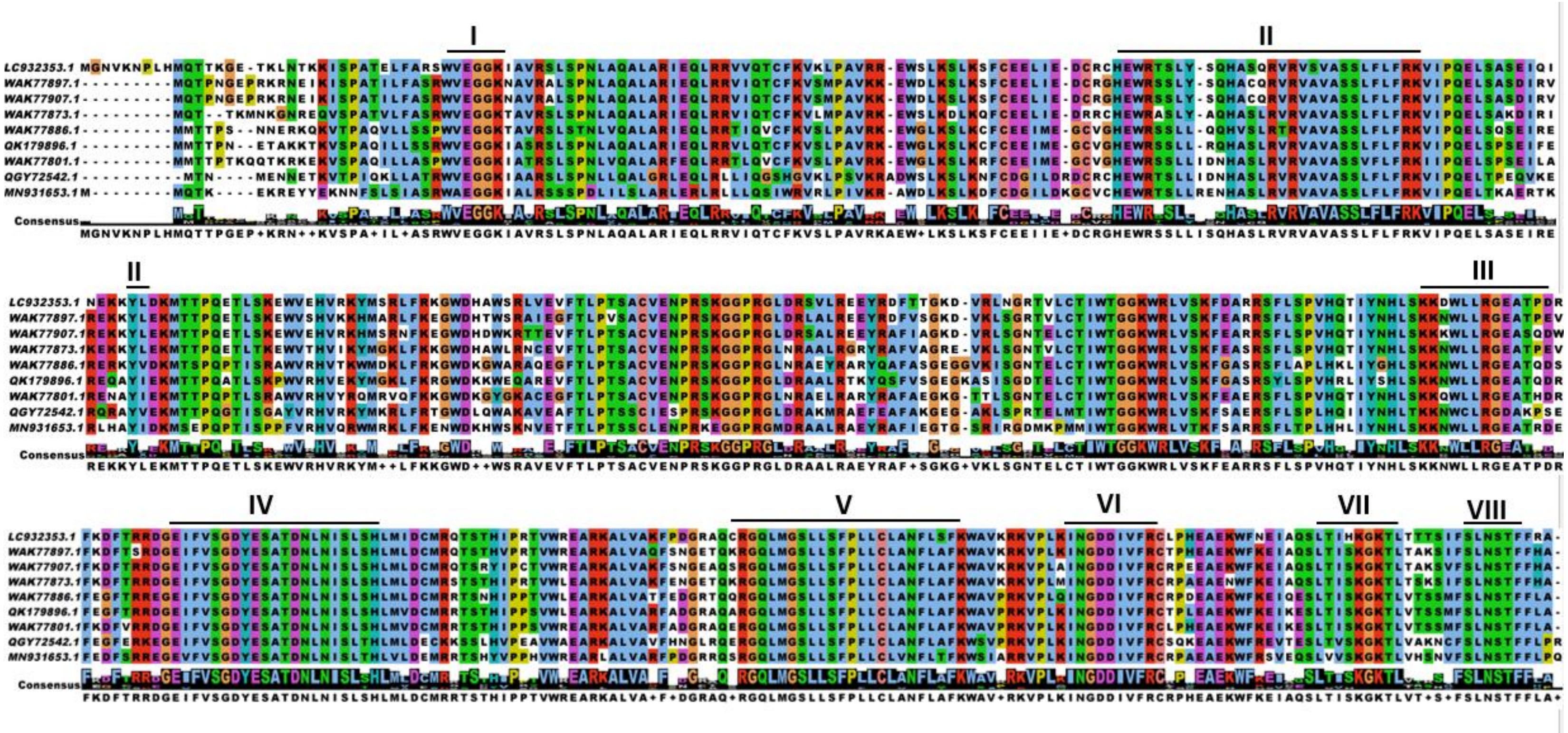

Figure S3. Multiple alignment of the RdRP aa sequences of LbBoV-3 and eight previously identified members of the family *Botourmiaviridae*. The eight conserved RdRP motifs are indicated by Roman numerals I to VIII with black lines.

Table S3. List of viruses used for phylogenetic analysis.

Accession number	Virus name
**QDB75006.1**	Acremonium sclerotigenum ourmia-like virus 1
**MZ600058.1**	Aspergillus flavus magoulivirus 1
**WAK77897.1**	Botourmiaviridae Dog tick metagenome isolate A
**WAK77907.1**	Botourmiaviridae Dog tick metagenome isolate B
**WAK77873.1**	Botourmiaviridae Dog tick metagenome isolate C
**WAK77886.1**	Botourmiaviridae Goat tick metagenome isolate D
**MN931653.1**	Botryosphaeria dothidea ourmia-like virus 1
**MN605470.1**	Botrytis cinerea ourmia-like virus 4
**QJT73671.1**	Botrytis cinerea ourmia-like virus 5
**MN605476.1**	Botrytis cinerea ourmia-like virus 10
**FJ157981.1**	Cassava virus C
**QDB75001.1**	Cladosporium uredinicola ourmia-like virus 1
**OQ162163.1**	Curvularia homomorpha ourmia-like virus 2
**QDB75003.1**	Epicoccum nigrum ourmia-like virus 1
**QDB75005.1**	Epicoccum nigrum ourmia-like virus 2
**EU770620.1**	Epirus cherry virus
**QGZ98425.1**	Erysiphe necator associated ourmia-like virus 5
**QK179896.1**	Erysiphe necator associated ourmia-like virus 66
**MN611654.1**	Erysiphe necator associated ourmia-like virus 127
**OP441679.1**	Leptosphaeria biglobosa botourmiavirus 1
**OP441680.1**	Leptosphaeria biglobosa botourmiavirus 2
**LC932353.1**	Leptosphaeria biglobosa botourmiavirus 3
**QLJ94076.1**	Magnaporthe oryzae botourmiavirus 5
**QLJ94430.1**	Magnaporthe oryzae botourmiavirus 6
**QLJ94431.1**	Magnaporthe oryzae botourmiavirus 7
**MT995746.1**	Magnaporthe oryzae botourmiavirus 9
**MK231023.1**	Monilinia ourmiavirus B
**B3VML1.1**	Ourmia melon virus
**AYP71797.1**	Penicillium citrinum ourmia-like virus 1
**QDB75000.1**	Penicillium sumatrense ourmia-like virus 1
**QDB75004.1**	Phaeoacremonium minimum ourmia-like virus 1
**QDB75007.1**	Phaeoacremonium minimum ourmia-like virus 2
**QDB75009.1**	Phaeoacremonium minimum ourmia-like virus 3
**QGY72531.1**	Plasmopara viticola lesion associated ourmia-like virus 1
**QGY72532.1**	Plasmopara viticola lesion associated ourmia-like virus 2
**QGY72534.1**	Plasmopara viticola lesion associated ourmia-like virus 4
**QGY72535.1**	Plasmopara viticola lesion associated ourmia-like virus 5
**WAK77801.1**	Plasmopara viticola lesion associated ourmia-like virus 8
**QGY72539.1**	Plasmopara viticola lesion associated ourmia-like virus 9
**QGY72541.1**	Plasmopara viticola lesion associated ourmia-like virus 11
**QGY72542.1**	Plasmopara viticola lesion associated ourmia-like virus 12
**MN532621.1**	Plasmopara viticola lesion associated ourmia-like virus 34
**MN532624.1**	Plasmopara viticola lesion associated ourmia-like virus 37
**MN532626.1**	Plasmopara viticola lesion associated ourmia-like virus 39

**MN532627**.1	Plasmopara viticola lesion associated ourmia-like virus 40
**QGY72574.1**	Plasmopara viticola lesion associated ourmia-like virus 44
**QGY72575.1**	Plasmopara viticola lesion associated ourmia-like virus 45
**QGY72576.1**	Plasmopara viticola lesion associated ourmia-like virus 46
**QGY72577.1**	Plasmopara viticola lesion associated ourmia-like virus 47
**QGY72579.1**	Plasmopara viticola lesion associated ourmia-like virus 49
**QGY72580.1**	Plasmopara viticola lesion associated ourmia-like virus 50
**QGY72582.1**	Plasmopara viticola lesion associated ourmia-like virus 52
**QGY72583.1**	Plasmopara viticola lesion associated ourmia-like virus 53
**QGY72585.1**	Plasmopara viticola lesion associated ourmia-like virus 55
**QGY72590.1**	Plasmopara viticola lesion associated ourmia-like virus 60
**QGY72594.1**	Plasmopara viticola lesion associated ourmia-like virus 64
**QGY72610.1**	Plasmopara viticola lesion associated ourmia-like virus 80
**QGY726213.1**	Plasmopara viticola lesion associated ourmia-like virus 83
**QGY726216.1**	Plasmopara viticola lesion associated ourmia-like virus 86
**QGY72620.1**	Plasmopara viticola lesion associated ourmia-like virus 90
**BBF90576.1**	Pyricularia oryzae ourmia-like virus 1
**OM514631.1**	Sanya botourmia-like virus 8
**ALD89138.1**	Sclerotinia sclerotiorum ourmia-like virus 1
**ALD89139.1**	Sclerotinia sclerotiorum ourmia-like virus 2
**AWY11006.1**	Sclerotinia sclerotiorum ourmia-like virus 3
**ALM62238.1**	Soybean leaf-associated ourmiavirus 1
**ALM622501.1**	Soybean leaf-associated ourmiavirus 2
**XFO98488.1**	unclassified magoulivirus (partial sequence)

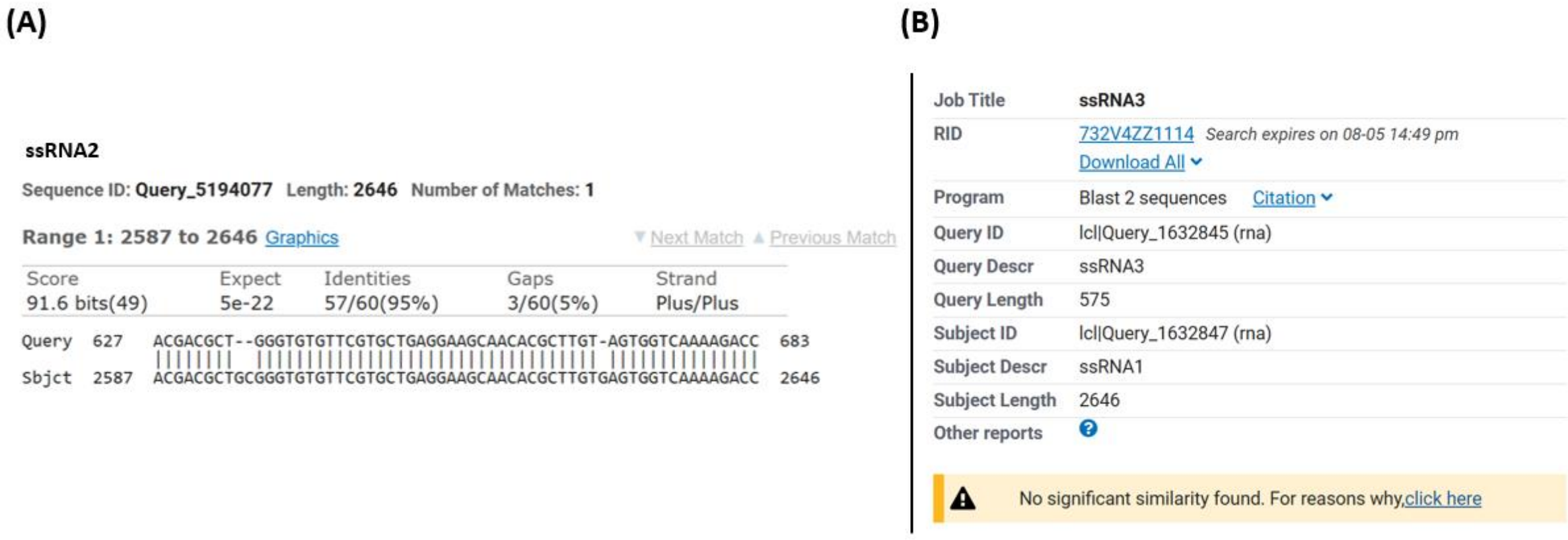

Figure. S4. Pairwise BLASTn analysis of the two satellite-like RNAs against the genomic RNA (ssRNA1) of LbBoV-3. **(A)** A single significant BLASTn hit was detected between ssRNA2 and the 3'-terminal region of the LbBoV-3 genomic RNA. **(B)** No significant nucleotide sequence similarity was detected between ssRNA3 and the LbBoV-3 genomic RNA.
